# Characterization of the Performance of an XXZ Three-Spin Quantum Battery

**DOI:** 10.3390/e27050511

**Published:** 2025-05-10

**Authors:** Suman Chand, Dario Ferraro, Niccolò Traverso Ziani

**Affiliations:** 1 Dipartimento di Fisica, Università di Genova, Via Dodecaneso 33, 16146 Genova, Italy; dario.ferraro@unige.it (D.F.); niccolo.traverso.ziani@unige.it (N.T.Z.); 2 CNR-SPIN, Via Dodecaneso 33, 16146 Genova, Italy

**Keywords:** quantum batteries, xxz spin chain, quantum phase transition

## Abstract

Quantum batteries represent a new and promising technological application of quantum mechanics, offering the potential for enhanced energy storage and fast charging. In this work, we study a quantum battery composed of three two-level systems with XXZ coupling operating under open boundary conditions. We investigate the role played by ferromagnetic and antiferromagnetic initial configurations on the charging dynamics of the battery. Two charging mechanisms are explored: static charging, where the battery interacts with a constant classical external field, and harmonic charging, where the field oscillates periodically over time. Our results demonstrate that static charging can be more efficient in the ferromagnetic case, achieving maximum energy due to complete population inversion between the ground and excited states. In contrast, harmonic charging excels in the antiferromagnetic case. By analyzing the stored energy and the average charging power in these two regimes, we highlight the impact of anisotropy on the performance of quantum batteries. Our findings provide valuable insights for optimizing quantum battery performance based on the system’s initial state and coupling configuration, paving the way for the study of more efficient quantum devices for energy storage.

## 1. Introduction

Quantum batteries (QBs) represent a cutting-edge approach to energy storage, leveraging quantum mechanical principles such as coherence and entanglement to achieve superior performance of devices over their classical counterparts [1,2,3,4]. The rapid advancement of quantum information science [5,6] and quantum thermodynamics [7,8,9,10,11,12] has led to an increased interest in exploring the advantages that QBs can offer, particularly in terms of short charging times and improved energy storage capacities [13,14,15,16,17,18,19,20,21,22,23,24,25,26,27,28,29,30,31,32,33,34,35,36,37].

Notable models considered for the realization of QBs are spin systems [38,39,40,41,42,43,44] where qubits, described as quantum spins, interact under different coupling mechanisms. This allows for the detailed investigation of relevant phenomena such as quantum phase transitions (QPTs) [45,46,47], which play a significant role in determining the time evolution and the performance of QBs [48] and, more generally, quantum thermal machines [49,50]. Studies have shown that the presence of QPTs can enhance energy storage by reorganizing the ground state of the system, thus impacting the overall charging dynamics [51,52,53,54]. This aspect is crucial for understanding how the ground state configuration of a spin system can be harnessed to optimize its performance as a QB [55].

In this direction, our study focuses on exploring a simple model of QB based on three spins in an XXZ configuration, where anisotropy in the interaction leads to distinct ferromagnetic and antiferromagnetic configurations for the ground state. We investigate the role played by these initial states on the performance of the QB by analyzing the stored energy and average charging power achieved during the charging process. Two distinct charging mechanisms are considered: static charging, where the system is subjected to a constant classical external field, and harmonic charging, where the charging field periodically oscillates over time. Our goal is to study the interplay between the ferromagnetic and antiferromagnetic phases, controlled through the anisotropy parameter Δ, and these two charging protocols to determine the more efficient charging for each initial condition. Throughout this work, we refer to the ground state as ferromagnetic-like or antiferromagnetic-like based on the dominant spin correlations [56,57]. We emphasize that these terms are used descriptively; indeed, in a three-spin system, they do not denote the true thermodynamic phases.

This work aims to provide insights into optimizing QB performance by properly tailoring the charging scheme to the system’s initial state. Our results demonstrate that while static charging performs better in the ferromagnetic case, harmonic charging excels in the antiferromagnetic case.

The paper is organized as follows: In Section 2, we describe the QB model, determine the initial conditions, and introduce the different charging mechanisms. In Section 3, we present the results and discuss the QB performance in various configurations. Finally, in Section 4, we provide the conclusions.

## 2. Model of the QB, Charging Schemes and Figures of Merit

### 2.1. Anisotropic XXZ Spin QB

**Figure 1:**
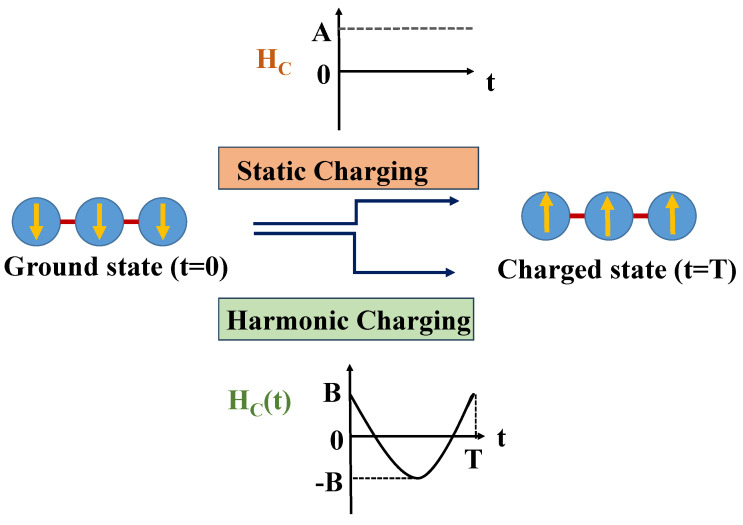
Schematic diagram displaying the charging scheme of the QB. When t=0, the system is in its ground state (ferromagnetic in the example shown). The QB undergoes charging via two schemes: static and harmonic charging. In both cases, the QB interacts with the charging field only for a given time 0<t<T, which can be modified to optimize the performance of the device.

We consider a QB consisting of a three-qubit anisotropic XXZ chain under the influence of a uniform external magnetic field *h*. The system operates under open boundary conditions, as illustrated in Figure 1. The Hamiltonian of the system is described (in units of Planck’s constant ℏ=1) as follows:

(1) $$H_{B}=-J\sum_{n=1}^{2}\left(S_{x}^{\left(n\right)}S_{x}^{\left(n+1\right)}+S_{y}^{\left(n\right)}S_{y}^{\left(n+1\right)}+\Delta S_{z}^{\left(n\right)}S_{z}^{\left(n+1\right)}\right)-2h\sum_{n=1}^{3}S_{z}^{\left(n\right)},$$

where $S_{\alpha }^{n}$ represents spin operators associated with the *n*-th site of the chain such that for the considered spin-1/2 system, $S_{\alpha }^{n}=\frac{1}{2}\sigma_{\alpha }^{n}$ with $\sigma_{\alpha }^{n}$ (α=x,y,z) being the conventional Pauli matrices. The magnetic field *h* acts along the *z*-axis, *J* represents the coupling strength between adjacent qubits, and Δ is the anisotropy parameter along the *z*-axis. For each spin, one can define a ground and an excited state with a spinorial form

$$|g\rangle =\left(\begin{array}{c} 1\\ 0 \end{array}\right),\;|e\rangle =\left(\begin{array}{c} 0\\ 1 \end{array}\right)$$

respectively. The eigenstates and eigenvalues of $H_{B}$ in the three-qubit bare basis $\left\{|ggg\rangle ,|gge\rangle ,|geg\rangle ,\ldots ,|eee\rangle \right\}$ can be expressed as follows:

(2) $$\begin{array}{ccc} |E_{1}\rangle & = & -|gee\rangle +|eeg\rangle ,\;E_{1}=h;\\ |E_{2}\rangle & = & |ggg\rangle ,\;E_{2}=-3h-\frac{\Delta J}{2},\\ |E_{3}\rangle & = & |eee\rangle ,\;E_{3}=3h-\frac{\Delta J}{2},\\ |E_{4}\rangle & = & -|gge\rangle +|egg\rangle ,\;E_{4}=-h,\\ |E_{5,6}\rangle & = & |gee\rangle +X_{1,2}|ege\rangle +|eeg\rangle ,\;E_{5,6}=\frac{\Delta J}{4}+h\mp 4JY_{1},\\ |E_{7,8}\rangle & = & |gge\rangle +X_{3,4}|geg\rangle +|egg\rangle ,\;E_{7,8}=\frac{\Delta J}{4}-h\mp 4JY_{1}, \end{array}$$

where the parameters $X_{1,2}$, $X_{3,4}$, and $Y_{1}$ are defined as follows:

(3) $$\begin{array}{ccc} X_{1,2} & = & \mp \frac{\sqrt{\Delta^{2}+8}}{2}+\frac{\Delta }{4}+\frac{4h}{J}\\ X_{3,4} & = & \pm \frac{\sqrt{\Delta^{2}+8}}{2}-\frac{\Delta }{4}\\ Y_{1} & = & \frac{1}{4}\sqrt{\frac{\Delta^{2}}{16}+\frac{1}{2}}. \end{array}$$

Notice that, for the sake of notational convenience, the above states are not normalized.

Assuming that *J* is the unit of energy from this point onward, the behavior of the system depends crucially on the anisotropy parameter Δ. Indeed, it is possible to determine a crossover value $\Delta_{C}$ such that for $\Delta >\Delta_{C}$, the system shows a ferromagnetic ground state, where all spins align along the same direction. Conversely, for $\Delta <\Delta_{C}$, the ground state of the system exhibits an antiferromagnetic behavior. This transition occurs at $\Delta =\Delta_{C}$, which separates the ferromagnetic and antiferromagnetic ground states and plays a major role in the energy storage capabilities of the present device, as will be clearer in the following.

Notice that due to the complexity of the calculations involved, these considerations, as well as the precise value of the crossover parameter $\Delta_{C}$, have been derived from a numerical analysis.

To clarify our terminology, we now explicitly state that $\Delta_{C}$ does not represent a critical value in the sense of a true phase transition but rather a crossover point in our finite three-spin system. In the thermodynamic limit (N→∞), the XXZ chain exhibits a genuine antiferromagnetic phase. However, in our small system with N=3, $\Delta_{C}$ marks a qualitative shift in ground-state properties. Specifically, we define $\Delta_{C}$ as the value of Δ at which the ground state undergoes an energy level crossing (from $E_{2}$ to $E_{7}$), transitioning from a ferromagnetic-like configuration, $|E_{2}\rangle$$\left(\Delta >\Delta_{C}\right)$, to a state exhibiting antiferromagnetic correlations, $|E_{7}\rangle$$\left(\Delta <\Delta_{C}\right)$. This crossover is further supported by the behavior of the nearest-neighbor spin correlation function and the antiferromagnetic structure factor, both of which exhibit a qualitative change around $\Delta_{C}$. To maintain consistency with the terminology used in larger systems, we retain the designation $\Delta_{C}$ while explicitly clarifying that it signifies a finite-size crossover rather than a true critical point.

### 2.2. Charging Protocols

In this section, we introduce two distinct charging mechanisms for the QB: static and harmonic charging. For both of them, we will study the time evolution of the QB and evaluate its stored energy and average charging power (see below). At time t=0, the QB is initialized in its ground state whose nature, as stated above, depends on the value of the anisotropy Δ. The system then evolves under the influence of a charging field up to a time t=T carefully chosen to reach a maximally charged state. The time evolution is unitary, and we will approach it in two different ways. We will first assume that the QB Hamiltonian $H_{B}$ is only used to fix the initial conditions of the system and that the unitary evolution is induced by a, possibly time-dependent, charging Hamiltonian $H_{C}\left(t\right)$ [58]. During this process, the battery Hamiltonian $H_{B}$ is therefore turned off to focus solely on the charging mechanism’s efficiency, inspired by similar approaches in two-level quantum systems like the Rabi model [39,59,60]. Specifically, the charging Hamiltonian $H_{C}$ is turned on at t=0, driving the system’s evolution and injecting energy into the battery. At a chosen time t=T, when the stored energy reaches a maximum, the charging Hamiltonian is turned off, and the system subsequently evolves again under the battery Hamiltonian $H_{B}$. After, we will consider a more experimentally feasible situation where the unitary evolution is induced by the total Hamiltonian $H_{B}+H_{C}\left(t\right)$. The precise expressions considered for $H_{C}\left(t\right)$ are discussed in the following, and the problem will be approached by numerically solving the Schödinger equations.

#### 2.2.1. Static Charging

The static charging Hamiltonian is given by the following:

(4) $$H_{C}=A\sum_{n=1}^{3}S_{x}^{\left(n\right)},$$

where *A* is the charging amplitude and $S_{x}^{\left(n\right)}$ are the spin operators along the *x* direction for the *n*-th spin of the chain.

#### 2.2.2. Harmonic Charging

In this case, the system is exposed to the periodic time-dependent driving field

(5) $$H_{C}\left(t\right)=B\cos\left(\omega t\right)\sum_{n=1}^{3}S_{x}^{\left(n\right)},$$

where *B* is the amplitude of the harmonic driving field and ω is the modulation frequency. Also, in this case, we assume that the harmonic charging field is applied up to a time t=T that needs to be chosen in such a way as to optimize the performance of the QB. This optimization will be discussed in the subsequent sections.

We choose the charging Hamiltonians $H_{C}=A\sum_{n}S_{x}^{\left(n\right)}$ (static) and $H_{C}\left(t\right)=B\cos\left(\omega t\right)\sum_{n}S_{x}^{\left(n\right)}$ (harmonic) representative forms of uniform driving acting on the spins, commonly used in the quantum battery literature [1,38,61,62,63,64]. These operators rotate all spins uniformly and can be implemented using standard transverse fields in trapped-ion or superconducting qubit systems [65,66], while they may not be optimal in a control-theoretic sense, they provide analytically tractable and physically realizable testbeds to assess the influence of anisotropy in the battery Hamiltonian $H_{B}$.

### 2.3. Stored Energy and Averaged Charging Power

The total energy stored in the QB at a given time *t*, indicated as E(t), is defined as the difference between the energy of the system in the evolved state and the initial state [16]:

(6) $$E\left(t\right)=\mathrm{Tr}\left(H_{B}\rho \left(t\right)\right)-\mathrm{Tr}\left(H_{B}\rho \left(0\right)\right),$$

where ρ(t) is the density matrix of the system at time *t*, with ρ(0) representing the initial state. This definition captures the energy absorbed from the charging process that is stored in the battery, relative to its ground-state energy. In our charging protocol, we consider two distinct approaches to unitary evolution. In the first, the battery Hamiltonian $H_{B}$ is used solely to define the initial state, and the system evolves under a charging Hamiltonian $H_{C}\left(t\right)$, which is externally switched on at t=0 and off at a chosen time t=T. In the second, more experimentally realistic scenario, the system evolves under the full Hamiltonian $H\left(t\right)=H_{B}+H_{C}\left(t\right)$ throughout the charging process. In both cases, energy is supplied to the system via the externally controlled charger $H_{C}\left(t\right)$. Even in the static case where $H_{C}$ is time-independent, its sudden activation at t=0 injects energy into the system. In the harmonic case, the time-dependent form of $H_{C}\left(t\right)$ provides continuous periodic energy injection. Although the evolution remains unitary, it is driven by external control, and energy is not conserved with respect to the battery Hamiltonian $H_{B}$. The stored energy E(t) quantifies the net energy absorbed by the system, using $H_{B}$ as the observable to measure useful energy. Since $H_{B}$ is not necessarily part of the time-evolution operator during charging, E(t) reflects the extractable energy once $H_{C}\left(t\right)$ is turned off. The corresponding average charging power for a given time *t* is given by the following:

(7) $$P\left(t\right)=\frac{E(t)}{t}.$$

The goal of the charging process is to choose the time t=T to maximize the stored energy or the maximum charging power. Notice that, in general, these two times do not coincide; therefore, the choice has to be made taking into account the specific figure of merit relevant to the actual purpose of the considered QB. In particular, the maximum average charging power, defined as the total stored energy divided by the elapsed time, occurs at an intermediate time when the energy storage rate is highest. Stored energy, however, continues to increase beyond this point, reaching its maximum at a later time. This discrepancy arises because average power reflects the average rate of energy storage over time, while stored energy reflects the total accumulated energy. Our results reflect this effect: average power peaks when the charging rate is highest, whereas energy reaches its maximum later as the system approaches saturation.

In this study, we have explored the role of initial conditions and charging protocols. However, optimizing charging time presents an interesting avenue for future research. A systematic analysis should investigate how charging time and power depend on the battery Hamiltonian $H_{B}$ parameters, ensuring that the ground state remains within the same phase (ferromagnetic or antiferromagnetic). This would help identify parameter regimes that enable faster charging or higher stored energy.

As stated above, the QB is initialized in the ground state of $H_{B}$, corresponding to a zero-temperature state. The effect of the initial state on the charging dynamics is highly dependent on the anisotropy parameter Δ, which controls the nature of the ground state. By comparing the performance of the quantum battery under the previously discussed charging schemes, we can analyze the role of the form of the initial state on the performance of the QB. This allows us to identify the optimal charging strategy based on the QB’s initial conditions and the specific charging protocol.

## 3. Charging Performance

In this section, we study the QB charging considering as initial state both the ferromagnetic and antiferromagnetic ground state (characterized by different values of the anisotropy Δ) and under the influence of the static and the harmonic charging field.

### 3.1. Static Charging with Ferromagnetic and Antiferromagnetic Initial State

We begin by considering the static charging, governed by the charging Hamiltonian $H_{C}=A\sum_{n=1}^{3}S_{x}^{\left(n\right)}$ (see Equation (4)), where we assume the driving amplitude A=1.

In this case, the charging process is described by the simple time evolution operator $U\left(t\right)=\exp\left(-iH_{C}t\right)$, with the QB Hamiltonian $H_{B}$ turned off during the charging process (see Section 2.2). Numerically, we find that for a coupling strength of J=1 and a magnetic field strength of h=1, the crossover value of the anisotropy parameter is $\Delta_{C}=-1.58$. According to this, we will study the QB ground state for two values of the anisotropy:

- $\Delta =-1.5>\Delta_{C}$: Ferromagnetic ground state.
- $\Delta =-1.6<\Delta_{C}$: Antiferromagnetic ground state.

#### 3.1.1. Ferromagnetic Ground State (Δ=−1.5):

Here, all spins are aligned in the same direction. Within the present choice of parameters, the initial energy of the battery in this configuration is $E_{\mathrm{Initial}}=-2.25$, corresponding to the state where all three spins are individually in their ground state. The battery is considered fully charged when all three spins are in their individually excited state, resulting in a final energy $E_{\mathrm{Final}}=3.75$. Therefore, the maximum energy that can be stored in the QB is as follows:

$$E_{\max}=E_{\mathrm{Final}}-E_{\mathrm{Initial}}=6.$$

**Figure 2:**
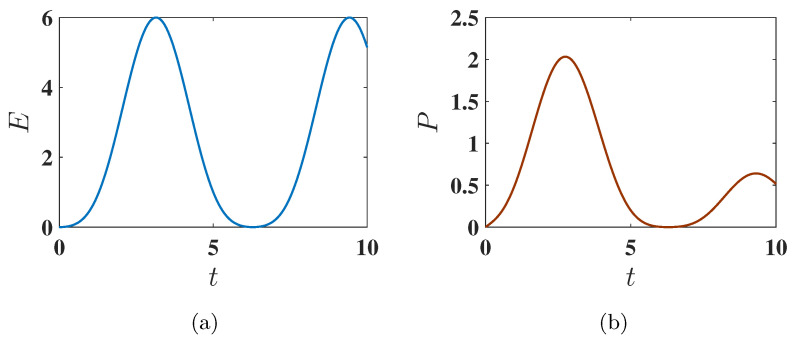
Behavior of (**a**) stored energy E(t) and (**b**) average charging power P(t) of the QB as a function of charging time *t* for Δ=−1.5, corresponding to the ferromagnetic ground state. The charging is performed using the static field $U\left(t\right)=\exp\left(-iH_{C}t\right)$. Other parameters used are J=1, h=1, and A=1.

**Figure 3:**
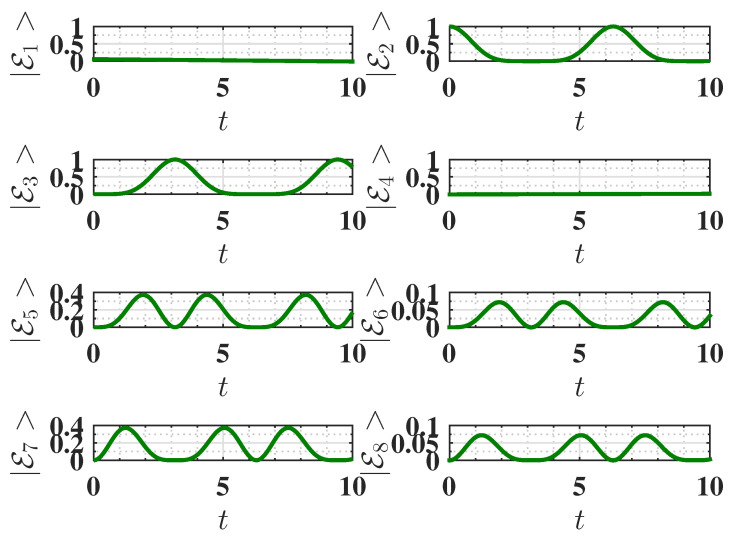
Behavior of the eigenstate populations (see Equation (2)) as a function of charging time *t* for Δ=−1.5, corresponding to the ferromagnetic ground state. The charging is performed using the static field $U\left(t\right)=\exp\left(-iH_{C}t\right)$. The parameters are the same as Figure 2. We recall that in our notation, $|E_{2}\rangle$ is the ground state, and $|E_{3}\rangle$ is the highest excited state (see Equation (2)).

As shown in Figure 2a, this maximum possible energy can indeed be achieved. This is due to the fact that the considered static charging leads to a complete population swap between the ground state ($|E_{2}\rangle =|ggg\rangle$) and the excited state ($|E_{3}\rangle =|eee\rangle$) of the system, as shown in Figure 3. This efficient population transfer allows the complete charge of the QB by applying a static field.

#### 3.1.2. Antiferromagnetic Ground State (Δ=−1.6):

In this regime, the spins are not individually in their ground states but instead start in a superposition state of the form

$$|\Psi \rangle =C_{1}|gge\rangle +C_{2}|geg\rangle +C_{3}|egg\rangle ,$$

where $C_{1}$, $C_{2}$, and $C_{3}$ are constants determined by the values of *J* and *h*. For the considered choice of parameters, one has $C_{1}=C_{3}=0.3562$, and $C_{2}=0.8638$, satisfying the constraint $C_{1}^{2}+C_{2}^{2}+C_{3}^{2}=1$ as expected. In this case, the initial energy of the battery is $E_{\mathrm{Initial}}=-2.2124$, and the final energy when fully charged is $E_{\mathrm{Final}}=3.8000$. Thus, the maximum energy that can be achieved is as follows:

$$E_{\max}=E_{\mathrm{Final}}-E_{\mathrm{Initial}}=6.0124.$$

Notice that this value crucially depends on the magnitude of the parameter Δ.

**Figure 4:**
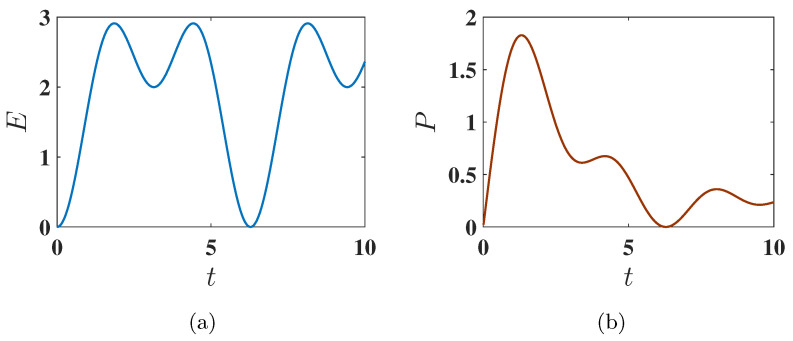
Behavior of (**a**) the stored energy E(t) and (**b**) the average power P(t) of the battery as a function of charging time *t* for Δ=−1.6, corresponding to the antiferromagnetic ground state. The charging is performed using the static charging field described by $U\left(t\right)=\exp\left(-iH_{C}t\right)$. Other parameters are the same as Figure 2.

**Figure 5:**
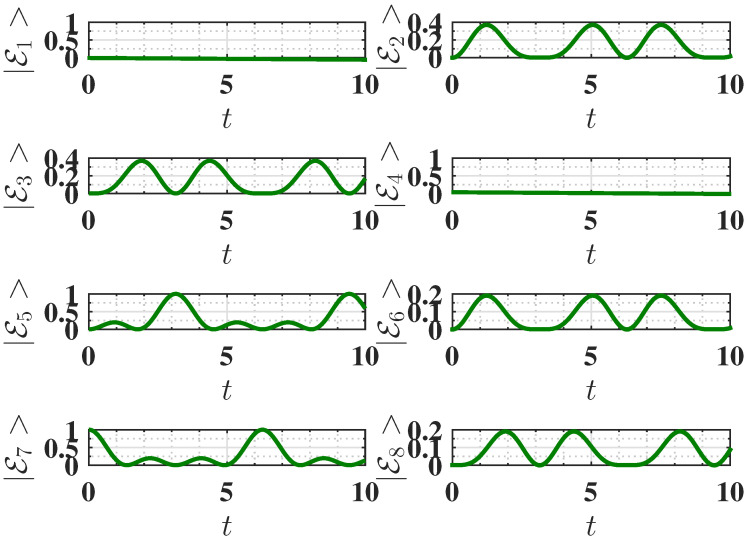
Behavior of the eigenstate populations (see Equation (2)) as a function of charging time *t* for Δ=−1.6, corresponding to the antiferromagnetic ground state. The charging is performed using the static field $U\left(t\right)=\exp\left(-iH_{C}t\right)$. Other parameters are the same as Figure 2. We recall that in our notation, $|E_{7}\rangle$ is the ground state, and $|E_{3}\rangle$ is the highest excited state (see Equation (2)).

As shown in Figure 4a, the QB initialized in the antiferromagnetic ground state can only reach roughly half of this maximum energy due to incomplete population transfer between the ground state $|E_{7}\rangle$ and the excited state $|E_{3}\rangle$. This incomplete population swap can be seen in Figure 5.

The efficiency of charging depends on the interplay between the battery Hamiltonian $H_{B}$ and the charging Hamiltonian $H_{C}$, while the antiferromagnetic initial state showed poor performances for our chosen $H_{C}$, we extend our discussion to different charging schemes based on theoretical considerations. Our findings suggest that an appropriately chosen $H_{C}$ can significantly enhance charging performance. In particular, a uniform transverse field appears to be the most effective charger across both ferromagnetic and antiferromagnetic states. The staggered field may perform comparably to the transverse field for ferromagnetic states but seems less effective for antiferromagnetic states. Meanwhile, a Heisenberg-type interaction is expected to be inefficient in both cases.

#### 3.1.3. Average Charging Power

Comparing the average charging power P(t) for the ferromagnetic and antiferromagnetic initial conditions, we observe that its maximum $P_{\max}$ is higher for the ferromagnetic ground state (see the comparison between Figure 2b and Figure 4b). However, interestingly, in the antiferromagnetic ground state, the maximum power is reached in a shorter time compared to the ferromagnetic ground state. This indicates that, for this initial condition, one has a faster but less complete population transfer.

### 3.2. Harmonic Charging with Ferromagnetic and Antiferromagnetic Initial State

In this subsection, we explore the performance of the QB under harmonic charging. The time-dependent Hamiltonian governing the charging process is $H_{C}\left(t\right)=B\cos\left(\omega t\right)\sum_{n=1}^{3}S_{x}^{\left(n\right)}$ (see Equation (5)), where we assume the driving amplitude B=1 and the modulation frequency of the harmonic field ω=1. The time evolution of the QB is then described by the unitary operator $U\left(t\right)=T\exp(-i\int_{0}^{t}H_{C}\left(t^{\prime }\right)dt^{\prime })$, where T indicates the time-ordering. Here, the QB Hamiltonian $H_{B}$ only imposes the initial conditions and is turned off during charging (see Section 2.2).

This protocol allows us to analyze how the oscillatory nature of the harmonic field impacts the QB’s charging efficiency for both ferromagnetic and antiferromagnetic initial states.

#### 3.2.1. Ferromagnetic Ground State (Δ=−1.5)

**Figure 6:**
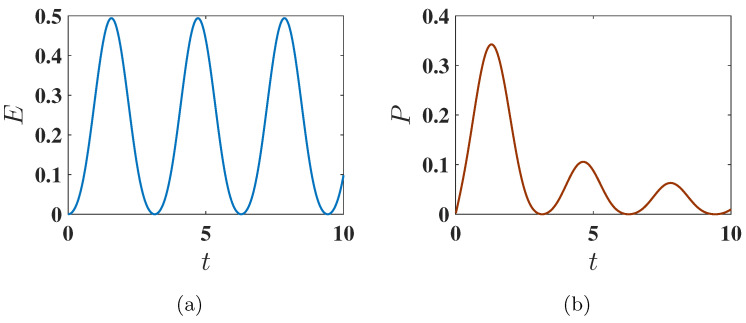
Behavior of (**a**) the stored energy E(t) and (**b**) the average charging power P(t) of the QB as a function of charging time *t* for Δ=−1.5, corresponding to the ferromagnetic ground state. The charging is performed using the harmonic charging field described by $U\left(t\right)=T\exp(-i\int_{0}^{t}H_{C}\left(t^{\prime }\right)dt^{\prime })$. Other parameters used are J=1, h=1, ω=1 and B=1.

**Figure 7:**
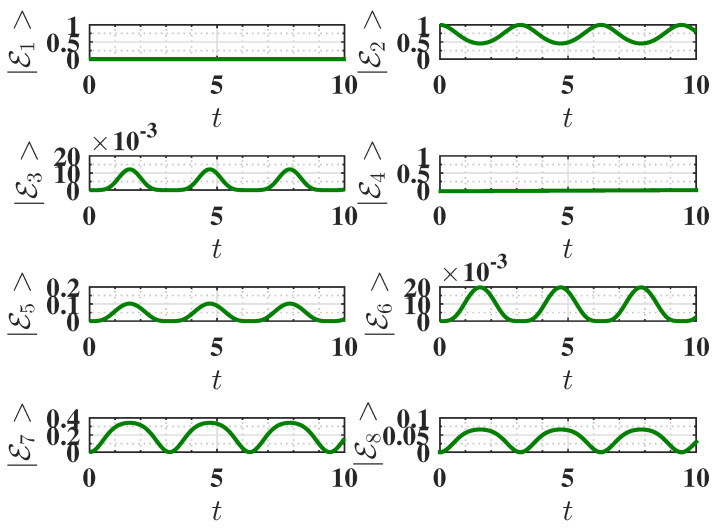
Behavior of the eigenstate populations (see Equation (2)) as a function of charging time *t* for Δ=−1.5, corresponding to the ferromagnetic ground state. The charging is performed using the harmonic field, with $U\left(t\right)=T\exp(-i\int_{0}^{t}H_{C}\left(t^{\prime }\right)dt^{\prime })$. The parameters are the same as Figure 6. We recall that in our notation, $|E_{2}\rangle$ is the ground state, and $|E_{3}\rangle$ is the highest excited state (see Equation (2)).

As discussed above, here we have $E_{\max}=6$. However, as shown in Figure 6a, the QB does not reach the maximum energy under harmonic charging due to the incomplete population transfer between the ground state ($|E_{2}\rangle =|ggg\rangle$) and the excited state ($|E_{3}\rangle =|eee\rangle$), as indicated by the eigenstate population dynamics shown in Figure 7. In this case, the oscillatory nature of the harmonic field disrupts the efficient population swap observed in static charging. Consequently, the QB achieves a lower energy output than the static field case.

#### 3.2.2. Antiferromagnetic Ground State (Δ=−1.6)

**Figure 8:**
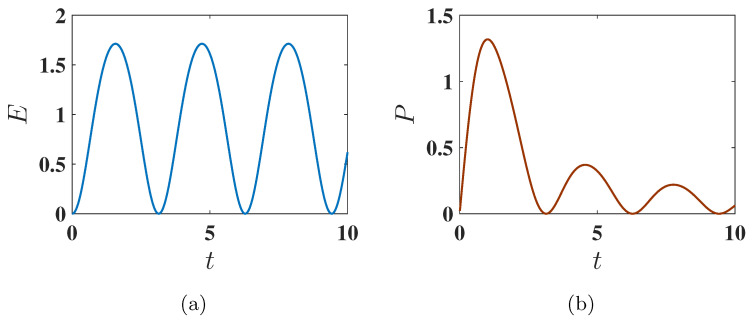
Behavior of (**a**) the stored energy E(t) and (**b**) the average charging power P(t) as a function of charging time *t* for Δ=−1.6, corresponding to the antiferromagnetic ground state. The charging is performed using the harmonic field, with $U\left(t\right)=T\exp(-i\int_{0}^{t}H_{C}\left(t^{\prime }\right)dt^{\prime })$. Other parameters are the same as Figure 6.

**Figure 9:**
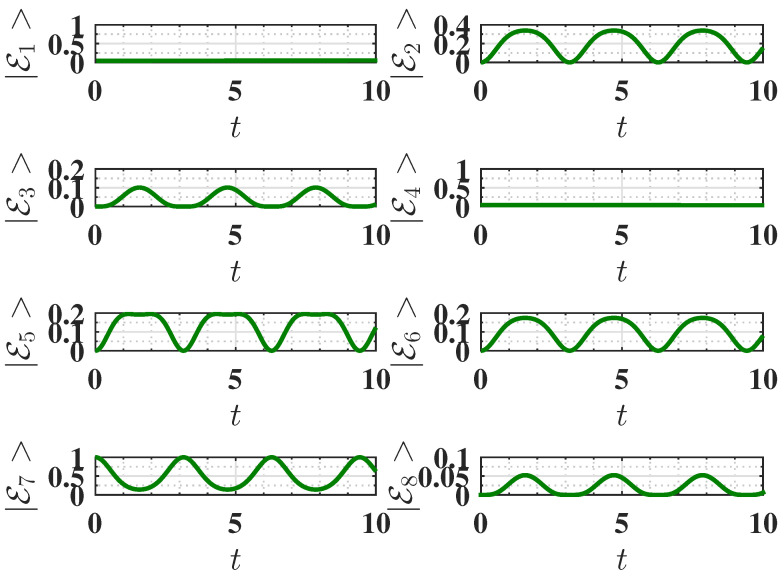
Behavior of the eigenstate populations (see Equation (2)) as a function of charging time *t* for Δ=−1.6, corresponding to the antiferromagnetic ground state. The charging is performed using the harmonic field, $U\left(t\right)=T\exp(-i\int_{0}^{t}H_{C}\left(t^{\prime }\right)dt^{\prime })$. Other parameters are the same as Figure 6. We recall that in our notation $|E_{7}\rangle$ is the ground state, and $|E_{3}\rangle$ is the highest excited state (see Equation (2)).

Here, the maximum energy is $E_{\max}=6.0124$. Interestingly, the antiferromagnetic state shows a more favorable performance under harmonic charging than the ferromagnetic state. As shown in Figure 8a, the energy stored starting from the antiferromagnetic ground state is significantly higher than in the ferromagnetic case. This improved performance is attributed to the more efficient population swap between the ground state ($|E_{7}\rangle$) and excited states (specifically the highest energy state $|E_{3}\rangle$), as indicated by the eigenstate population dynamics in Figure 9.

The improved performance of harmonic charging in the antiferromagnetic case arises from the fundamental difference in the interaction structure of the spin chain. In a ferromagnetic system, spins naturally align in the same direction, so applying a uniform charging protocol to all spins is relatively efficient. However, in an antiferromagnetic chain, adjacent spins tend to align oppositely, and because of this, applying the same charging field uniformly to all spins is not the most efficient way to transfer energy. The introduction of an oscillatory (harmonic) charging field provides a new timescale in the system, which enables a resonant enhancement of energy transfer between competing spin configurations. In contrast, for the ferromagnetic case, the system is already aligned, so the introduction of periodic driving does not play a crucial role. This suggests that harmonic charging is particularly beneficial when spin interactions create an energetically unfavorable configuration for uniform charging.

The energy storage dynamics for different values of the driving field amplitude *B* reveal a fundamental difference between the ferromagnetic and antiferromagnetic systems. In the ferromagnetic case, energy absorption remains low for small B≪ω, as the driving field weakly perturbs the system. In contrast, the antiferromagnetic system exhibits efficient energy absorption even at small *B*, likely due to enhanced spin correlations that facilitate energy transfer. As *B* increases, both systems show an increase in energy storage, but the antiferromagnetic case consistently outperforms the ferromagnetic one. These findings highlight the role of intrinsic spin interactions in determining the efficiency of quantum battery charging under harmonic driving.

**Figure 10:**
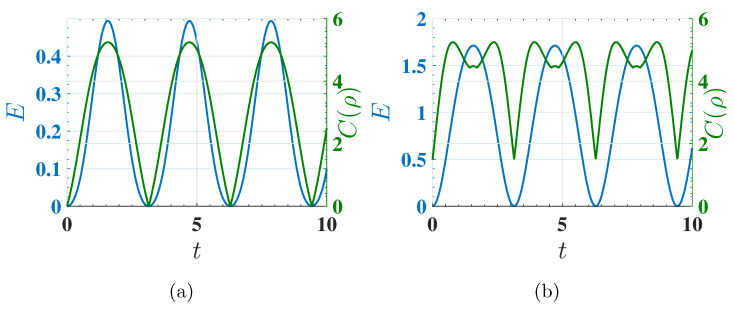
The behavior of the stored energy E(t) (left *y*-axis) and $l_{1}$-norm coherence C(ρ) (right *y*-axis) for (**a**) the ferromagnetic case (Δ=−1.5) and (**b**) the antiferromagnetic case (Δ=−1.6) as a function of charging time *t*. These plots illustrate how coherence affects energy storage dynamics, where charging is performed using the harmonic field described by $U\left(t\right)=T\exp(-i\int_{0}^{t}H_{C}\left(t^{\prime }\right)dt^{\prime })$. Sustained coherence in the antiferromagnetic case correlates with higher energy storage, whereas transient coherence in the ferromagnetic case limits energy transfer. Other parameters are the same as in Figure 6.

To further characterize the charging process, we investigate the role of quantum coherence as a potential figure of merit for energy storage. Specifically, we analyze the time evolution of the $l_{1}$-norm coherence:

$$C\left(\rho \right)=\sum_{i\neq j}\left|\rho_{ij}\right|$$

where $\rho_{ij}$ are the off-diagonal elements of the density matrix. This measure quantifies the total amount of quantum superposition in the system. We compare the behavior of stored energy and coherence under harmonic driving for ferromagnetic (Δ=−1.5) and antiferromagnetic (Δ=−1.6) cases in Figure 10, which presents the time evolution of stored energy E(t) and coherence C(ρ). In the ferromagnetic case, coherence builds up transiently but is rapidly lost, leading to poor energy storage. By contrast, in the antiferromagnetic case, coherence is sustained throughout the charging process, facilitating stronger energy storage. These results suggest that coherence plays a crucial role in energy transfer, with persistent coherence corresponding to more efficient quantum charging. The findings highlight the importance of long-lived coherence rather than transient effects, reinforcing the role of quantum correlations in energy storage.

#### 3.2.3. Maximum Average Power

Comparing Figure 6b and Figure 8b, we observe that under harmonic charging, the maximum average power $P_{\max}$ is significantly higher in the antiferromagnetic case compared to the ferromagnetic one. Interestingly, also for this protocol, $P_{\max}$ is reached faster starting from the antiferromagnetic state than from the ferromagnetic state.

### 3.3. Joint Evolution Considering QB and Charging Hamiltonians

We present here the results of charging the QB through the joint evolution of the battery Hamiltonian $H_{B}$ and the charging Hamiltonian $H_{C}$.

#### 3.3.1. Static Charging

**Figure 11:**
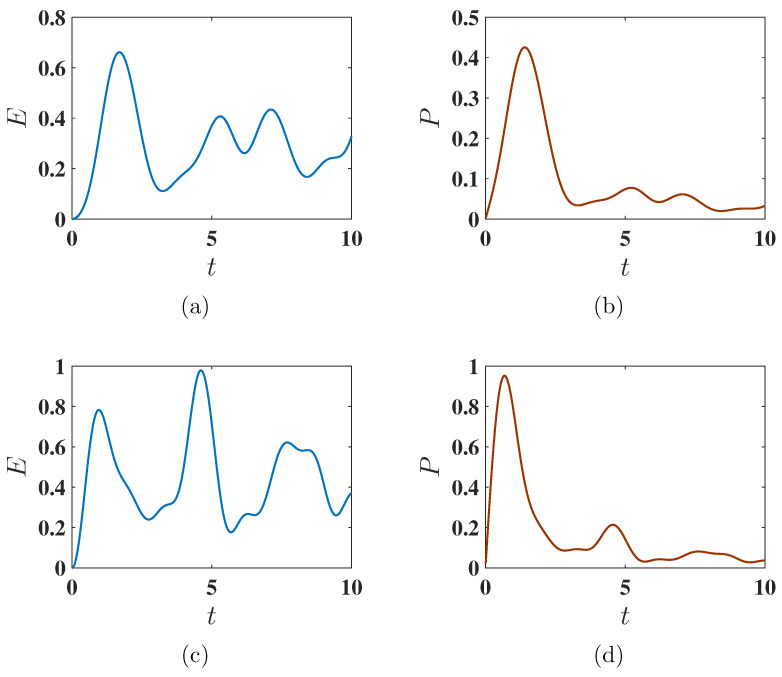
Behavior of the stored energy E(t) and average charging power P(t) as functions of charging time *t* under the static charging protocol. Panels (**a**,**b**) correspond to the ferromagnetic initial state with Δ=−1.5, while panels (**c**,**d**) correspond to the antiferromagnetic initial state with Δ=−1.6. The charging is performed using the static field $U\left(t\right)=\exp(-i\left(H_{B}+H_{C}\right)t)$. Other parameters are set to J=1, h=1, and A=1.

We consider here the case where the charging process occurs in the presence of both the QB Hamiltonian $H_{B}$ and the charging Hamiltonian $H_{C}$, i.e., the unitary operator leading to the evolution of the initial state of the QB is given by $U\left(t\right)=\exp(-i\left(H_{B}+H_{C}\right)t)$ using static charging method (see Equation (4)). The time evolution of the stored energy E(t) and average power P(t) for both the ferromagnetic and antiferromagnetic initial states under joint evolution is shown in Figure 11. In this scenario, the QB does not achieve the maximum available energy. This can be attributed to the poorer population swap between the ground and excited states. Nonetheless, in the case of the antiferromagnetic initial state, the maximum power $P_{\max}$ is still reached faster than in the ferromagnetic state.

#### 3.3.2. Harmonic Charging

**Figure 12:**
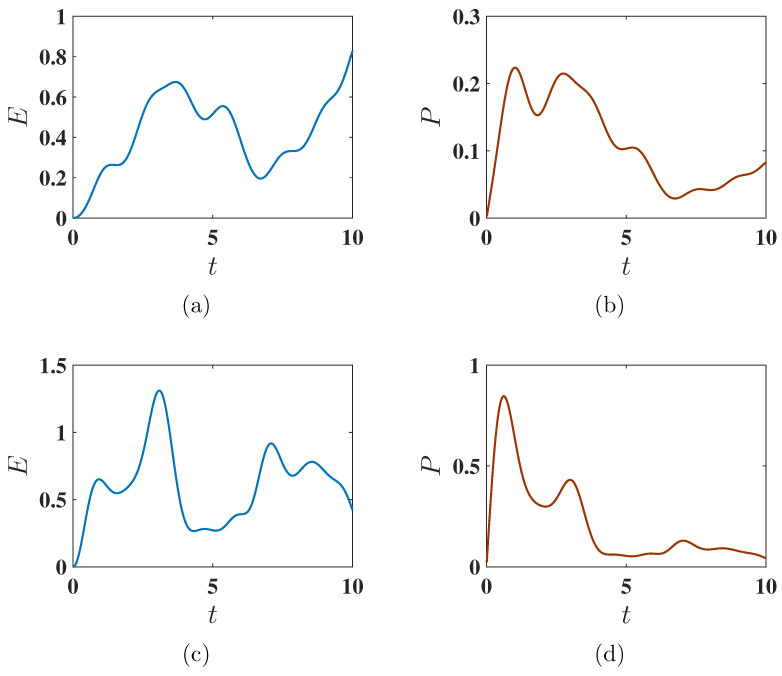
Behavior of the stored energy E(t) and average charging power P(t) as functions of charging time *t* under the harmonic charging method. Panels (**a**,**b**) correspond to the ferromagnetic initial state with Δ=−1.5, while panels (**c**,**d**) correspond to the antiferromagnetic initial state with Δ=−1.6. The charging is performed using the harmonic field described by $U\left(t\right)=T\exp\left(-i\int_{0}^{t}\left[H_{B}+H_{C}\left(t^{\prime }\right)\right]dt^{\prime }\right)$. Other parameters are set as J=1, h=1, ω=1, and B=1.

Here, the initial state of the QB evolves in time according to both $H_{B}$ and $H_{C}\left(t\right)$, with time evolution governed by the unitary operator $U\left(t\right)=T\exp\left(-i\int_{0}^{t}\left[H_{B}+H_{C}\left(t^{\prime }\right)\right]dt^{\prime }\right)$, where T indicates the time ordering. In Figure 12, we display the variation of the stored energy E(t) and average power P(t) for the harmonic charging method (Equation (5)). Similarly to the static case, the QB does not reach maximum charging. However, the charging speed improves, especially in the antiferromagnetic state, where $P_{\max}$ is reached faster. Overall, joint evolution allows faster charging compared to the isolated charging field alone, but at the cost of reduced energy output. Interestingly, the antiferromagnetic case consistently shows faster charging compared to the ferromagnetic one.

## 4. Conclusions

In this work, we investigated the charging performance of a quantum battery composed of three qubits, modeled as a spin system with XXZ coupling. By considering both static and harmonic charging mechanisms, we observed distinct behaviors depending on the nature of the initial condition, namely ferromagnetic or antiferromagnetic, achieved by moving the anisotropy parameter Δ above and below a numerically determined crossover value $\Delta_{C}$, respectively.

Starting from a ferromagnetic ground state, characterized by $\Delta >\Delta_{C}$, the system exhibits well-aligned spins, allowing efficient population transfer between the ground state $|E_{2}\rangle$ and the excited state $|E_{3}\rangle$. Under static charging, the system reaches its maximum achievable energy, as reflected in the stored energy and average charging power. In contrast, harmonic charging under these conditions fails to reach the same energy levels due to incomplete population transfer caused by the oscillatory nature of the driving field.

In the antiferromagnetic case, where $\Delta <\Delta_{C}$, static charging struggles to achieve high energy levels, whereas harmonic charging performs significantly better. The oscillatory field facilitates population transfer, resulting in higher stored energy and faster charging times. These findings suggest that the choice of charging mechanism should be tailored to the initial state of the quantum battery to optimize its performance.

Our analysis also reveals that quantum coherence serves as a meaningful figure of merit for characterizing quantum battery charging for harmonic charging. The ferromagnetic case (Δ=−1.5) exhibits transient coherence that quickly disappears, leading to poor energy storage. In contrast, the antiferromagnetic case (Δ=−1.6) maintains more stable coherence, which correlates with higher energy absorption and better charging performance. These findings suggest that the sustained presence of coherence, rather than its instantaneous peak, plays a crucial role in enhancing quantum battery efficiency.

We also analyzed the more realistic joint evolution of the QB under the combined influence of the QB Hamiltonian and the charging Hamiltonian for both static and harmonic fields. In this case, we observed that while the quantum battery does not reach its maximum energy capacity, the charging process is accelerated in the antiferromagnetic case, highlighting the role of anisotropy in optimizing charging efficiency. This effect is likely due to the competition between spin interactions, which enhances the system’s ability to absorb energy over shorter time periods, albeit at the cost of reduced overall energy output.

We have focused on a small system size N=3, to explore the fundamental role of initial conditions and charging protocols. However, the scaling of maximum charging energy with system size is an important question in spin-based quantum batteries, and a detailed numerical study of this effect in our system represents an important future development of this work. Moreover, we have highlighted the importance of the initial conditions in determining the optimal charging strategy for QB. Future research could explore more complex spin systems or alternative coupling mechanisms, which may reveal additional quantum advantages in energy storage.

## Data Availability

The data presented in this study are available on request from the corresponding author.

## Abbreviations

The following abbreviations are used in this manuscript:

QB	Quantum battery
QPT	Quantum phase transition
